# Supplementary material for: Anti-Cancer Effect of Cordycepin on FGF9-Induced Testicular Tumorigenesis
Source: Int J Mol Sci. 2020 Nov 6;21(21):8336. doi: 10.3390/ijms21218336 (PMC7672634; doi:10.3390/ijms21218336)
Supplement: Supplementary file 1 [file ijms-21-08336-s001.zip › Table S2 Antibodies used in this study.pdf]

Table S2. Antibodies used in this study.

Antibody	Manufacturer [a]	Catalogue No.	Species/Clonality	Application [b]
**Primary Antibody**				
β-actin	Sigma-Aldrich	A5441	mouse/monoclonal	WB
CDK1	AbCam	ab18	mouse/monoclonal	WB
CDK2	Cell Signaling	#2546	rabbit/monoclonal	WB
CDK4	AbCam	ab137675	rabbit/monoclonal	WB
Cleaved-Caspase 3	Cell Signaling,	#9661	rabbit/polyclonal	WB, IHC
Cyclin A1	Proteintech	13295-1-AP	rabbit/monoclonal	WB
Cyclin B1	Abcam	ab181593	rabbit/monoclonal	WB
Cyclin D1	Cell Signaling	#2978	rabbit/polyclonal	WB
Cyclin E1	Cell Signaling	#20808	rabbit/polyclonal	WB
E2F1	Abcam	ab179445	rabbit/monoclonal	WB
	Abcam	ab112580	rabbit/polyclonal	IP, ChIP
	Proteintech	12171-1-AP	rabbit/polyclonal	IHC
ERK1/2	Cell Signaling	#9102	rabbit/polyclonal	WB, IHC
Phosphor-ERK1/2	Cell Signaling	#9101	rabbit/polyclonal	WB
	Abcam	ab201015	rabbit/monoclonal	IHC
FGF9	Abcam	ab9743	rabbit/polyclonal	WB, IHC
FGFR1	Sigma-Aldrich	F5421	rabbit/polyclonal	WB, IF
FGFR2	Sigma-Aldrich	F6769	rabbit/polyclonal	WB
	Abcam	ab10648	rabbit/polyclonal	IF
FGFR3	Sigma-Aldrich	F3922	rabbit/polyclonal	WB
	Abcam	ab180906	rabbit/polyclonal	IF
FGFR4	Abcam	ab5481	rabbit/polyclonal	WB, IF
IgG	Jackson ImmunoResearch	005-000-003	goat	Negative control for all experiments
		011-000-003	rabbit	
Ki-67	Abcam	ab16667	rabbit/polyclonal	WB, IHC
mTOR	Cell Signaling	#2983	rabbit/polyclonal	WB
Phosphor-mTOR	Cell Signaling	#2971	rabbit/polyclonal	WB
p21	GeneTex	GTX62525	rabbit/polyclonal	WB
Phosphor-p21	Santa Cruz Biotechnology	sc-20220-R	rabbit/polyclonal	WB
p27	Cell Signaling	#3698	rabbit/polyclonal	WB
Phosphor-p27	Cell Signaling	#2404	rabbit/polyclonal	WB
p53	Cell Signaling	#2524	rabbit/polyclonal	WB
Phosphor-p53	Cell Signaling	#9284	rabbit/polyclonal	WB
PLCγ1	Cell Signaling	#5690	rabbit/polyclonal	WB
phosphor-PLCγ1	Cell Signaling	#2821	rabbit/polyclonal	WB
Rb	Abcan	Ab181616	rabbit/monoclonal	WB
P-Rb	Cell Signaling	#8516	rabbit/monoclonal	WB, IHC
**Secondary Antibody**				
Alexa Flour® 488-conjugated anti-rabbit IgG	Invitrogen / Thermo Fisher Scientific	A11008	Donkey	IF
Anti-mouse IgG HRP-conjugated	PerkinElmer	NEF82200-1EA	Goat	WB
Anti-rabbit IgG HRP-coniugated	PerkinElmer	NEF81200-1EA	Goat	WB

[a] The headquarters locations of companies: Abcam, Cambridge, UK; Cell Signaling, Beverly, MA, USA; GeneTex, Irvine,

CA, USA; Invitrogen, Waltham, MA, USA; Jackson ImmunoResearch, West Grove, PA, USA; PerkinElmer, Waltham, MA, USA; Proteintech, Rosemont, IL, USA ; Santa Cruz Biotechnology, Santa Cruz, CA, USA; Sigma-Aldrich, St. Louis, MO, USA; Thermo Fisher Scientific Inc., Waltham, MA, USA. [b] ChIP: Chromatin Immunoprecipitation; IF, immunofluorescence staining; IP: Immunoprecipitation; IHC, immunohistochemistry staining; WB, western blot analysis; HRP, horseradish peroxidase.
