# Supplementary material for: Anti-Cancer Effect of Cordycepin on FGF9-Induced Testicular Tumorigenesis
Source: Int J Mol Sci. 2020 Nov 6;21(21):8336. doi: 10.3390/ijms21218336 (PMC7672634; doi:10.3390/ijms21218336)
Supplement: Supplementary file 1 [file ijms-21-08336-s001.zip › Table S1 Chemicals and materials used in this study.pdf]

Table S1. Chemicals and materials used in this study.

Chemical / Material.	Manufacturer[a]	Catalogue No.
2-ME (2-Mercaptoethanol)	Alfa Aesar / Thermo Fisher Scientific	A15890
Acrylamide/Bis-acrylamide, 30% solution	Sigma-Aldrich	A3574-100ML
Agar	BD	214530
Agarose	GeneDirex	MB755-0100
Bovine serum albumin (BSA)	UniRegion BioTech	UR-BSA001-100G
Citric Acid	J.T. Baker/Avantor	0122-01
Cordycepin	Sigma-Aldrich	C3394-25MG
DAB (Mouse/Rabbit Probe HRP Labeling Kit)	BioTnA	TAHC03D-100
DMEM/F12 Dulbecco's modified eagle medium/ nutrient mixture F-12	Gibco / Thermo Fisher Scientific	12400-024
DMSO	Sigma-Aldrich	D4540-500ML
DPX Mountant for histology	Sigma-Aldrich	06522-100ML
ECL detection kit (Immunobilon® chemiluminescent HRP substrate)	Millipore	WBKLS0500
EDTA	Sigma-Aldrich	E5134-100G
EGTA	Merck	L808635342
Ethanol	J.T. Baker/Avantor	8006-05
Fetal bovine serum (FBS)	Gibco / Thermo Fisher Scientific	10437-028
FGF9, human, recombinant	PEPROTech	100-23
Glycine	J.T. Baker/Avantor	4059-06
Hematoxylin	Merck	HX68297174
HEPES [4-(2-hydroxyethyl)-1-piperazinee-thanesulfonic acid]	Sigma-Aldrich	H4034-100G
Hydrochloric acid (HCl)	Merck	K49418617738
LB Broth	BD	244620
Magna ChIP™ G Kit	EMD Millipore	MAGNA0002
Methanol	DUKSAN	62
MTT [3-(4,5-dimethylthiazol-2-yl)-2,5-diphenyltetrazolium bromide]	Sigma-Aldrich	M5655
Paraformaldehyde (PFA)	Merck	1,04005.1000
PD98059 (MAPK/ERK1/2 inhibitor)	Sigma-Aldrich	P215-5MG
Penicillin/Streptomycin	Simply	CC502-0100
Potassium chloride (KCl)	Sigma-Aldrich	P5405-250G
Potassium dihydrogen phosphate (KH_2PO_4)	Sigma-Aldrich	P5655-100G
ProLong® Diamond Antifade Mountant with DAPI	Thermo Fisher Scientific	P36962
Protease Inhibitor Cocktail	Sigma-Aldrich	P8340-1ML
Protein Assay Dye	BioRad Laboratories	5000006
Protein G magnetic Beads (PureProteome™)	EMD Millipore	LSKMAGG02
Puromycin	Sigma-Aldrich	P8833-100MG
Sodium bicarbonate ($NaHCO_3$)	Sigma-Aldrich	S5761-500G
Disodium hydrogen phosphate (Na_2HPO_4)	Sigma-Aldrich	S5136-100G

Table S1 *Cont.*

Sodium azide	Sigma-Aldrich	S2002-100G
Sodium chloride (NaCl)	Sigma-Aldrich	S6191-1KG
Sodium chloride (NaCl)	J.T. Baker/Avantor	3624-05
Sodium dodecyl sulfate (SDS)	SERVA	20765.03
Sodium hydroxide (NaOH)	Sigma-Aldrich	S2770
Sodium orthovanadate	Sigma-Aldrich	S6508
Sodium pyrophosphate	Riedel-de Haën	30411
TBE buffer (5x)	UniRegion Bio Tech	UR-TBEL
Tris base	J.T. Baker / Avantor	4109-06
Tris HCl	J.T. Baker / Avantor	4103-02
Triton X-100 (for IHC)	BDH laboratory Supplies	306324N
Triton X-100 (for cell lysate)	GERBU	2000
Trypen blue	Sigma-Aldrich	T8154
Trypsin-EDTA (0.5%, 10x)	Gibco / Thermo Fisher Scientific	15400-054
Tween 20	PanReac AppliChem	123412-1611
Waymouth MB 752/1 medium	Sigma-Aldrich	W1625-1L
Xylene	J.T. Baker / Avantor	9490-03
PVDF membrane (Polyvinylidence difluoride membrane; 0.45 micron pore size)	PALL Life Science	BSP0161

[a] The headquarters locations of companies: BD (Becton, Dickinson and Company), Franklin Lakes, NJ, USA; BioTnA, Kaohsiung, Taiwan; BDH laboratory Supplies, Kampala, Uganda; DUKSAN, Ansan City, Kyunggi, Korea; GeneDirex, Taichung, Taiwan; Gibco, Grand Island, NY, USA; GERBU Biotechnik GmbH, Heidelberg, Germany; J.T.Baker/Avantor Performance Materials, Center Valley, PA, USA; Merck, Darmstadt, Germany; Millipore, Billerica, MA, USA; EMD Millipore, Billerica, MA, USA; PALL Corporation, Port Washington, NY, USA; PanReac AppliChem, Darmstadt, Germany; PerkinElmer, Waltham, MA, USA; PEPROTech, Rocky Hill, NJ, USA; Riedel-de Haën/Honeywell, Morristown, NJ, USA; Sigma-Aldrich, St. Louis, MO, USA; SERVA Electrophoresis GmbH, Heidelberg, Germany; Thermo Fisher Scientific Inc., Waltham, MA, USA; UniRegion Bio Tech, Taipei, Taiwan.
